# Prevalence of circulating antibodies against hemagglutinin of influenza viruses in epidemic season 2021/2022 in Poland

**DOI:** 10.3389/abp.2024.12289

**Published:** 2024-02-19

**Authors:** Katarzyna Kondratiuk, Ewelina Hallmann, Karol Szymański, Katarzyna Łuniewska, Anna Poznańska, Lidia B. Brydak

**Affiliations:** ^1^ Laboratory of Influenza Viruses and Respiratory Viruses, Department of Virology, National Institute of Public Health NIH—National Research Institute, Warsaw, Poland; ^2^ Department of Population Health Monitoring and Analysis, National Institute of Public Health NIH—National Research Institute, Warsaw, Poland

**Keywords:** influenza, hemagglutinin antibodies, protection rate, GMT, serum financial support

## Abstract

The aim of the study was to determine the level of anti-hemagglutinin antibodies in the serum of patients during the 2021/2022 epidemic season in Poland. A total of 700 sera samples were tested, divided according to the age of the patients into 7 age groups: 0–4 years of age, 5–9 years of age, 10–14 years of age, 15–25 years of age, 26–44 years of age, 45–64 years of age and ≥65 years of age, 100 samples were collected from each age group. Anti-hemagglutinin antibody levels was determined using the haemagglutination inhibition assay (OZHA). The results obtained confirm the presence of anti-hemagglutinin antibodies for the antigens A/Victoria/2570/2019 (H1N1) pdm09, A/Cambodia/e0826360/2020 (H3N2), B/Washington/02/2019 and B/Phuket/3073/2013 recommended by World Health Organization (WHO) for the 2021/2022 epidemic season. The analysis of the results shows differences in the levels of individual anti-hemagglutinin antibodies in the considered age groups. In view of very low percentage of the vaccinated population in Poland, which was 6.90% in the 2021/2022 epidemic season, the results obtained in the study would have to be interpreted as the immune system response in patients after a previous influenza virus infection.

## Introduction

Influenza is a significant public health threat both in Poland and around the world. It is an acute infectious respiratory viral disease caused by influenza viruses classified into four types: A, B, C, and D. Influenza A virus infects not only humans, but also horses, pigs, aquatic mammals (such as seals, whales), and birds. The influenza B virus only infects humans, while the influenza C virus infects humans and pigs. In contrast, the influenza D virus has so far been detected in pigs and cattle (Ducatez et al., 2015; Gliński and Żmuda, 2022). Human infection with influenza D has not been observed (Wu and Wilson, 2020). This classification is based on antigenic differences between the main proteins of the virion, i.e., the M protein and the NP nucleoprotein. These types differ epidemiologically (Brydak, 2008). Influenza epidemics are caused by both influenza A (A/H3N2/and A/H1N1/pdm09) and influenza B (B/Yamagata and B/Victoria) lineages, but only influenza A viruses can cause a pandemic and dramatically increase hospitalizations and deaths from post-flu complications (Frey et al., 2023). In most cases, the influenza C virus causes only mild symptoms after infection.

Seasonal influenza mainly occurs during the colder months in regions with temperate climates. Human-to-human transmission of the influenza virus occurs through inhalation of infectious respiratory particles when an infected person coughs or sneezes. There is also evidence that the influenza virus can be transmitted by talking or breathing, that is by transmitting small respiratory particles. The incubation period of the influenza virus is usually 24–48 h. An infected patient is contagious even one to 2 days before the onset of influenza symptoms and for five to 7 days after the onset of symptoms. Infected children and persons taking immunosuppressive drugs may show prolonged secretion of the influenza virus (Gaitonde et al., 2019).

The influenza A virus particle, which deceptively resembles a chestnut with protruding spikes, has a lipid envelope originating from the plasma membrane of the host cell. Two surface proteins are anchored in it: hemagglutinin (HA) and neuraminidase (NA). Hemagglutinin is responsible for the adsorption of the influenza virus into the cell, while neuraminidase is responsible for the release of viruses from the host cells. It is estimated that there are approximately 400 HA spikes and 100 NA spikes per influenza A and B virus particle. The structure of the influenza virus genome is segmented. Influenza A and B viruses are characterized by having 8 viral RNA segments, while the influenza C virus has only 7 viral RNA segments. Influenza C viruses have only one type of spike in the lipid envelope, which serves as hemagglutinin and neuraminidase. The organization of the influenza A virus genome in the form of segments allows for reassortment, which is an important mechanism for the formation of diverse strains (Gliński and Żmuda, 2022). Thus, the influenza A virus is characterized by high antigenic variability, and based on differences in surface antigens, 11 subtypes conditioned by neuraminidase–NA (N1-N11) and 18 subtypes conditioned by hemagglutinin–HA (H1-H18) are distinguished (Brydak, 2008; Wierzbicka-Woś et al., 2015). Pandemic influenza strains arise as a result of antigenic shift and may include avian-derived hemagglutinin (HA) subtypes (such as H5, H7, and H9) or porcine hemagglutinin variants (H1, H2, and H3) which, by acquiring further adaptive mutations, become capable of human-to-human transmission of infection (Bouvier and Palese, 2008; Taubenberger and Kash, 2010; Khalenkov et al., 2023). Vaccination against influenza is the most effective preventive measure. Influenza vaccines reduce the risk of being affected by influenza and prevent the development of serious complications. They also play an important role in pandemic preparedness plans around the world (Frey et al., 2023).

However, in order to be effective, it requires constant and thorough worldwide supervision and timely updates of the formulation of the influenza vaccine (Khalenkov et al., 2023). Influenza virus infections, like many other infections, induce an immune response in the infected body, reducing or even cessating virus replication and formation of so-called immunological memory in the patient, protecting against subsequent infection. However, this optimistic scenario is hampered by the fact that the influenza A virus is characterized by high antigenic variability and constant mutations. This significantly impedes the formation of memory and immune response, while facilitating the transmission of infections between sick and healthy population (Wierzbicka-Woś et al., 2015). Antigenic drift (antigenic shift) is a consequence of point mutation of genes in the replication of influenza viruses, leading to changes in amino acid sequences that alter antigenic sites in epitopes. New variants and annual influenza epidemics are the result of antigenic changes in the H and N glycoproteins of the virus. This results in the inability to develop a universal formulation of the vaccine and the need to change the composition of influenza vaccines every season (Shao et al., 2017; Gliński and Żmuda, 2022). Anti-hemagglutinin antibodies are characterized by a relatively short persistence in blood serum (Allwinn et al., 2013). Due to all these reasons, seasonal influenza vaccinations are extremely important, contributing to an increase in resistance to influenza infections of the general population, and thus reducing the risk of post-influenza complications and death.

The aim of this study was to determine the level of anti-hemagglutinin antibodies in the sera of persons in different age groups during the 2021/2022 epidemic season in Poland.

## Materials and methods

The study material consisted of 700 sera collected by employees of 16 Provincial Sanitary and Epidemiological Stations in Poland, in accordance with the World Health Organization (WHO) recommendations. Samples were collected at voivodeship sanitary and epidemiological stations (VSES) in Poland, between 1st October 2021 and 31st September of 2022. The serum samples were divided into 7 groups according to the age of the patients: 0–4 years of age, 5–9 years of age, 10–14 years of age, 15–25 years of age, 26–44 years of age, 45–64 years old and ≥65 years of age, 100 samples collected from each age group (the number of samples from each voivodeship was not the same). Prior to testing, serum samples were stored at −80°C. Prior the testing sera were selected and checked for haemolysis in sample. If noticed, sample was discarded.

All viruses were obtained from World Influenza Centre at Francis Crick Institute, London, and then propagated in the amniotic cavity on 11- days old chicken embryos in Influenza Virus Research Department, National Influenza Center at the National Institute of Public Health NIH-National Research Institute (NIPH NIH-NRI), in accordance with WHO recommendations (WHO, 2011). The eggs were incubated as follows: for H3N2 and H1N1 viruses (2 days at 37°C); and for influenza B viruses (3 days at 35°C). Then titer of each virus was determined. Labelled vials containing viruses were stored at −80°C upon using in research (WHO, 2011).

The study used antigens for the 2021/2022 epidemic season recommended by WHO (Table 1).

**TABLE 1:** Influenza virus strains used for the hemagglutination inhibition assay (HAI) in the 2021/2022 epidemic season.

Epidemic season 2021/2022		
Influenza virus strains	A/H1N1/pdm09	A/Victoria/2570/2019 (H1N1)pdm09-like virus
	A/H3N2/	A/Cambodia/e0826360/2020 (H3N2)-like virus
	B Victoria lineage	B/Washington/02/2019 (B/Victoria lineage)-like virus
	B Yamagata lineage	B/Phuket/3073/2013 (B/Yamagata lineage)-like virus

The level of anti-hemagglutinin antibodies was determined by hemagglutination inhibition assay (HAI). The haemagglutination inhibition assay (OZHA) was performed using 8 hemagglutination units of the virus. The sera were inactivated prior to testing according to adopted standards (Tyrell and Horsfall et al., 1952; WHO, 2011). Necessary viruses with high titer were selected to be used in this test. Solution of titer 1:8 from each of the virus was prepared. For example, for virus titer 1:64, suspension of the virus was diluted 8 times. For 1:16—two Times. After preparing all necessary solution, they were stored in 4°C upon adding them on the plates.

PBS and Alsever’s solution needed for the OZHA test are prepared in–house. In this study V-bottom, clear, microtitration plates were used.

For OZHA test are used chicken red blood cells. Blood cells delivered to the laboratory were suspended in Alsever’s solution. By centrifugation at a speed of 1200 RPM for 10 min, a concentrate of packed blood cells was obtained and used in further research.

Each of the sera was treated with Receptor Destroying Enzyme (RDE) (Thermo Fisher Scientific) for 16 h at 37°C prior to the hemaggluti-nation inhibition test. After this step, to inac-tivate the enzyme, the mixture was incubated at 56°C for 30 min.

In OZHA test, a serial dilution of each of the sera was made in PBS. Then, the prepared solution of the virus, which has titer of 1:8 was added to each well on the plate. After virus addition, the plate was incubated for 15 min at room temperature. After incubation, 50 µL of blood cell solution was added. Readings were taken after 30 min of incubation at room temper-ature, then the results were read.

The analysis of the test results of the study was based on the following parameters: geometric mean titer (GMT) of the anti-hemagglutinin antibody in the tested sera and the protection rate (percentage of people with anti-hemagglutinin antibodies at a level ≥1:40 that appeared after the administration of an influenza vaccine or during the previous infection by the influenza virus) (Brydak, 2008; WHO, 2011; Krammer, 2019). This is the value of anti-hemagglutinin antibody titers, which is considered a protective value (Brydak, 2008).

### Statistical analysis

The test chi-square was used to compare age groups regarding the categorical variables (prevalence of anti-hemagglutinin antibodies and reaching their protective titer). The Kruskal-Wallis test was applied to compare the titer distribution between seven age groups, and the Mann-Whitney *U* test was used for two. The significance level for all the tests was assumed to amount to 0.05. The calculations were executed with the SPSS 12.0 PL.

## Results

The analysis of the obtained results shows that antibodies against A/Victoria/2570/2019 (H1N1) pdm09 were present in 332 people (47.4% of the studied patients). The largest number of tested patients—649 persons (92.7% of all subjects)—had antibodies against A/Cambodia/e0826360/2020 (H3N2). The smallest number of tested patients had antibodies against B/Washington/02/2019 (B/Victoria lineage), it was 288 patients (41.1% of subjects). Antibodies against B/Phuket/3073/2013 (B/Yamagata lineage) were present in the sera of 534 patients (76.3% of all tested sera).

**FIGURE 1:**
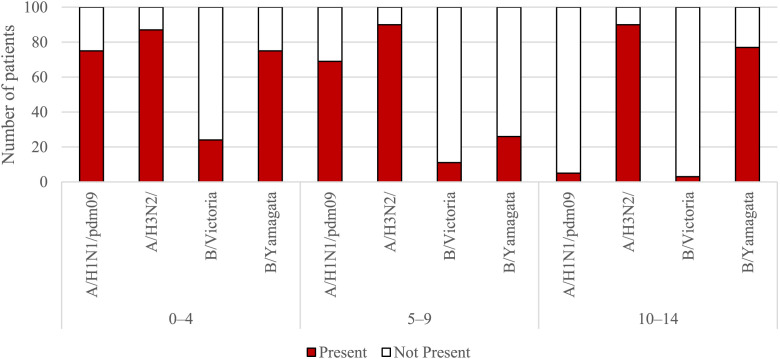
The presence of antibodies in the serum of patients aged 0–4 years, 5–9 years of age and 10–14 years of age in the 2021/2022 epidemic season.

**FIGURE 2:**
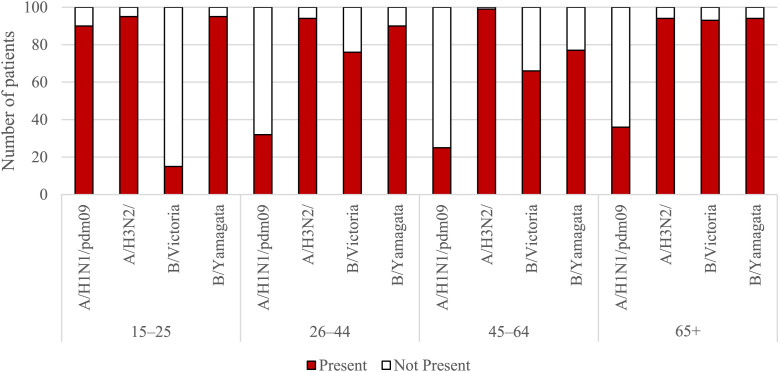
The presence of antibodies in the serum of patients aged 15–25, 26–44, 45–64, and 65+ years of age in the 2021/2022 epidemic season.

Figures 1, 2 show the percentage of patients in individual age groups that had antibodies against a particular influenza virus.

Using the chi-square test, it was found that statistically significant differences in the number (and therefore percentage) of those with antibodies between the tested sera of patients from all seven age groups apply to all four types of antibodies for the following types and subtypes of influenza viruses:

• For the H1 subtype: *p* < 0.001, the percentage of those with antibodies ranged from 5% in the 10–14-year-old group to 90% in the 15–25-year-old group;
• For the H3 subtype: *p* = 0.024, the percentage of those with antibodies ranged from 87% in the 0–4 years old group to 99% in the 45–64 years old group;
• For the B/Victoria line (B/Washington): *p* < 0.001, the percentage of those with antibodies ranged from 3% in the group of 10–14 years old to 93% in the group of 65 years and over;
• For the B/Yamagata line (B/Phuket): *p* < 0.001, the percentage of those with antibodies ranged from 26% in the 5–9 years old group to 95% in the 15–25 years old group.

**FIGURE 3:**
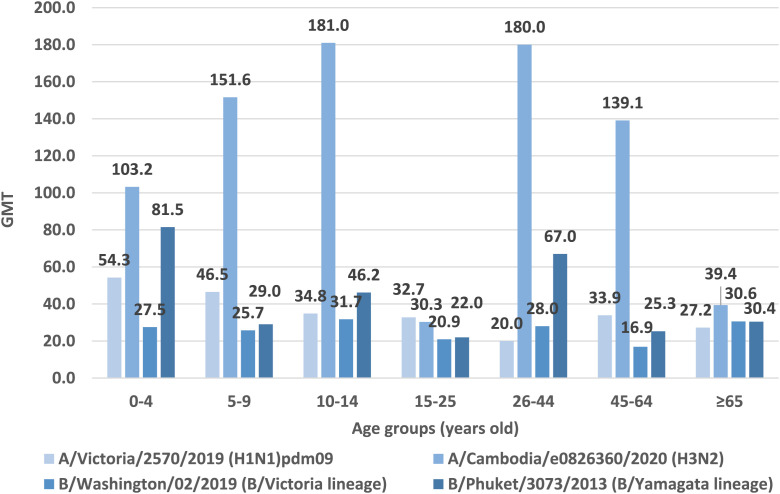
Geometric mean titers of anti-haemagglutinin antibodies (GMT) in the epidemic season 2021/2022 in age groups in Poland.

In Figure 3 shows the geometric mean titers of anti-hemagglutinin antibodies in the sera of patients according to age groups in the 2021/2022 epidemic season in Poland (Figure 1).

Based on the obtained test results, the highest GMT values for hemagglutinin A/H1 were found in patients in the youngest patients’ age group 0–4 years old (GMT = 54.3). Lower GMT values for hemagglutinin A/H1 were obtained in the 5–9 age group (GMT = 46.5). In the remaining age groups, GMT values for hemagglutinin A/H1 were comparable, with the lowest value in the age group of 26–44 years (GMT = 20.0).

The highest GMT values for hemagglutinin A/H3 was recorded in the age groups 10–14 years (GMT = 181.0) and 26–44 years (GMT = 180.0). Lower values were found in the age group of the youngest children aged 5–9 (GMT = 151.6), in patients aged 45–64 years (GMT = 139.1) and in the youngest patients’ age group 0–4 years old (GMT = 103.2). In the remaining patients, the GMT values for hemagglutinin A/H3 remained at a similar level: in the oldest patients over 65 years of age was 39.4 and in patients aged 15–25 was 30.3.

In the case of type B/Washington/02/2019, the highest GMT value was reported in the 10–14 age group (GMT = 31.7). In the remaining age groups, GMT values were as follows: ≥65 years old (GMT = 30.6), 26–44 years old (GMT = 28.0), 0–4 years old (GMT = 27.5), 5–9 years old (GMT = 25.7), 15–25 years old (GMT = 20.9), 45–64 years old (GMT = 16.9).

According to the analyzed data for type B/Phuket/3073/2013, the highest geometric mean antibody titers was reported for the youngest patients aged 0–4 years (GMT = 81.5). The age group 26–44 had a similar GMT value (GMT = 67.0). For the remaining age groups, the GMT values were: 10–14 years old (GMT = 46.2), ≥65 years old (GMT = 30.4), 5–9 years old (GMT = 29.0), 45–64 years old (GMT = 25.3), 15–25 years old (GMT = 22.0).

In neither age group the geometric mean titer of all of anti-hemagglutinin antibodies (H1, H3, B/Washington/02/2019 and B/Phuket/3073/2013) was not ≥ 40. In two age groups 15–25 and 65+, the average level of none of the antibodies reached 40. Only in the case of the youngest patients aged 0–4 years, the geometric mean titer of three anti-hemagglutinin antibodies (H1, H3 and B/Phuket/3073/2013) was ≥40. GMT values were respectively: for H3 GMT = 103.2, for B/Phuket/3073/2013 GMT = 81.5, for H1 GMT = 54.3, respectively.

Statistical analysis performed using the Kruskal-Wallis test showed that for all types of antibodies, the difference in their titer between all seven age groups was statistically significant (*p* < 0.001 in all cases).

• For the H1 subtype: antibodies found in 332 persons, the mean GMT = 37.0, the lowest antibody level in the group of 26–44 years old (GMT = 20.0), the highest in the group of 0–4 years old–GMT = 54.3 (p < 0.001);
• For the H3 subtype: antibodies found in 649 persons, the mean GMT = 97.1, the lowest antibody level in the group of 15–25 years old (GMT = 30.3), the highest in the group of 10–14 years old–GMT = 181.0 (p < 0.001);
• For the B/Victoria line (B/Washington): antibodies found in 288 persons, the mean GMT = 25.2, the lowest antibody level in the group of 45–64 years (GMT = 16.9), the highest in the group of 10–14 years–GMT = 31.8 (p < 0.001);
• For the B/Yamagata (B/Phuket) line: antibodies found in 534 persons, the mean GMT = 38.9, the lowest antibody level in the 15–25 years old group (GMT = 22.0), the highest in the 0–4 years old group–GMT = 81.5 (p < 0.001).

**FIGURE 4:**
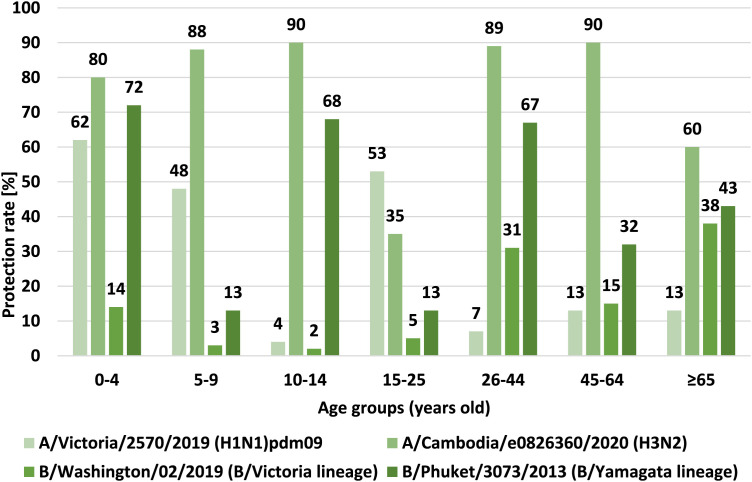
Percentage of cases with a protective titer of anti-haemagglutinin antibodies (%) in the 2021/2022 epidemic season in different age groups.

Figure 4 shows the percentage of cases with a protective anti-hemagglutinin antibody titer (%), i.e., ≥40, in the 2021–2022 epidemic season, in different age groups.

Studies on the effectiveness of influenza vaccination show that the protective coefficient should reach different values depending on the age of the patient in order to achieve the desired effect of vaccination effectiveness. According to the criteria of the CPMP (Committee for Proprietary Medicinal Products) of the European Agency for the Evaluation of Medicinal Products (EMEA) and the Commission of the European Communities regarding the harmonization of requirements for influenza vaccination, parameters such as the mean increase in anti-HA antibody titers (MFI), the protective factor and the response rate, measured approximately 3 weeks after vaccination should be taken into account when assessing the serological response to this vaccination. Depending on the age of the patient these values vary (Commission of the European Communities, 1991; Committee for Proprietary Medicinal Products, 1997).

Comparing the results of the protective coefficient results for all hemagglutinins, the highest protective coefficient values were obtained for hemagglutinin H3 and B/Yamagata (B/Phuket) line. The conducted analyzes of the value of the protective coefficient indicate that in the age group of 0–4, 5–9, 10–14, 26–44 and 45–64 years values above 70% and 60% for the 65+ age group have been recorded. These values were obtained only for the above mentioned subtypes. In other cases, the protection rate did not reach the recommended protection level. The highest value of protective coefficient was recorded for hemagglutinin H3. These data correspond to the low level of vaccination of the population in Poland in the 2021/2022 epidemic season (according to data from the Influenza Research Institute, National Influenza Centre).

For all antibody types, there is a statistically significant difference in protective coefficients between all seven age groups, as was demonstrated with the use of the chi-square test.

• For the H1 subtype: 200 subjects had antibodies with titers of ≥40, the protective coefficient was 29%, the statistical significance of differences between age groups was p < 0.001, the protective coefficients in age groups ranged from 4% in the group of 10–14 years old to 62% in the group of 0–4 years old;
• For the H3 subtype: 532 subjects had antibodies with titers ≥40, the protective coefficient was 76%, the statistical significance of differences between age groups was p < 0.001, the protective coefficients in age groups ranged from 35% in the group of 15–25 years old to 90% in groups of 10–14 years old and 45–64 years old;
• For the B/Victoria line (B/Washington): 108 subjects had antibodies with titers ≥40, the protective coefficient was 15%, statistical significance of differences between age groups was p < 0.001, protective coefficients in age groups from 2% in the group 10–14 years old to 38% in the group 65 years old and over;
• For the B/Yamagata (B/Phuket) line: 308 subjects had antibodies with titers ≥40, the protective coefficient was 44%, statistical significance of differences between age groups was p < 0.001, protective coefficients in age groups from 13% in groups of 5–9 years old and 15–25 years old to 72% in the 0–4 years old group.

Distributions of antibody levels among the tested sera of children (300 subjects aged 0–14) and adults (400 subjects aged over 14) show clear differences:

• For the H1 subtype: in adults, antibody titer values ranged from 0 to 640, quartiles (Q1/Q2—median/Q3): 0/0 (median)/20; in children values ranged from 0 to 160, quartile: 0/0 (median)/40; the difference in distributions is statistically significant (p = 0.002), as determined using the Mann-Whitney *U* test; the percentages of adults and children with antibodies are similar—45% vs. 50%, the difference is not statistically significant, as determined by the chi-square test; the mean level of antibodies in subjects who have them is significantly higher among children (49.8 vs. 29.1, p < 0.001), as shown by the Mann-Whitney *U* test; as expected (in both compared groups a similar percentage of subjects have antibodies, and their titers are clearly higher in younger ones), children reach the protective level significantly more often (38% vs. 22%; p < 0.001);
• For the H3 subtype: in both age groups, antibody titer values ranged from 0 to 640; quartile among adults: 20/80 (median)/160, higher among children 80/160 (median)/320; the difference in distributions is statistically significant (p < 0.001)—Mann-Whitney *U* test; presence of antibodies against H3 is common, 96% in adults and 89% in children, however the difference is statistically significant (*p* = 0.001, chi-square test); the mean level of antibodies (in people who have them) is significantly higher in children (142.0 vs 74.4; *p* < 0.001—Mann-Whitney *U* test); children are also a group that achieves the protective level significantly more often (86% vs. 69%; *p* < 0.001);
• For the B/Victoria line (B/Washington): in adults, antibody titer values ranged from 0 to 640, quartile: 0/10 (median)/20; in children, antibody titer values ranged from 0 to 160, quartile 0/0 (median)/0; the difference is statistically significant (*p* < 0.001)—Mann-Whitney *U* test; 63% of adults and only 13% of children had antibodies against B/Washington, the difference is statistically significant (*p* < 0.001, chi-square test); unlike other antibodies, there is no difference in their mean level in children (27.3) and adults (24.9)—as before, we only count subjects with antibodies; as expected (antibodies in adults are present much more often, and their average titer in both groups is similar), adults reach the protective level significantly more often (22% vs. 6%; *p* < 0.001);
• For the B/Yamagata (B/Phuket) line: in both age groups, antibody titer values ranged from 0 to 640; quartile in adults: 10/20 (median)/40, and in children 0/40/80; difference in distributions bordering on statistical significance (*p* = 0.053)—Mann-Whitney *U* test; 89% of adults and 59% of children had antibodies against B/Phuket, the difference is statistically significant (*p* < 0.001, chi-square test); the mean level of antibodies (in subjects with antibodies) is significantly higher in children (54.8 vs 32.7; *p* < 0.001—Mann-Whitney *U* test). The frequency of reaching the protective level is higher among children under 14 years of age (51% vs. 39%, *p* = 0.001).

## Discussion

The analysis of the study results indicates that antibodies against all four influenza viruses included in the influenza vaccine in the 2021/2022 influenza season were detected in the tested sera of patients from all age groups. Due to the very low vaccination rate of general population in Poland, which in the 2021/2022 epidemic season, despite recommendations and solid, multi-threaded educational campaigns, was estimated at only 6.90% (Grohskopf et al., 2021; NIPH NIH-NRI, 2023) the results obtained in the study should be interpreted as a response of the immune system in patients after a previous infection caused by the influenza virus. The detection of anti-hemagglutinin antibodies in the patient’s blood serum may indicate a past infection with influenza viruses or the fact that the patient has been vaccinated against influenza. Immunity after vaccination against influenza usually appears approximately 10–14 days after receiving the vaccine and lasts for approximately 6–12 months, i.e., during one epidemic season, which usually covers period from October to March. For this reason, and because the influenza virus is subject to rapid antigenic changes, seasonal influenza vaccination is extremely important. Vaccination is the most effective preventive measure against influenza infection, mitigating the course of the disease and preventing post-influenza complications, which may be very serious, including complications with death threat. This may not only apply to people in high-risk groups.

Comparing the antigenic composition of the influenza vaccine in effect in the Northern Hemisphere in the 2021/2022 epidemic season and in the 2020/2021 epidemic season, it can be noted that in both epidemic seasons the same antigens for type B influenza viruses were present: B/Washington/02/2019 (B/Victoria lineage)-like virus and B/Phuket/3073/2013 (B/Yamagata lineage)-like virus. In these two epidemic seasons, only the antigens for influenza A viruses mutated, both for the influenza virus of the A/H1N1/pdm09 subtype and for the A/H3N2/virus. The influenza B/Phuket/3073/2013 (B/Yamagata lineage)-like virus antigen has been included in the influenza vaccine recommended for the Northern Hemisphere for several consecutive epidemic seasons. Despite this, this virus reached a protective level only in one age group among the analyzed samples–among the youngest children under 4 years of age (72%).

It is considered necessary to vaccinate 70%–80% of the population to achieve community immunity. It is assumed that the titer of anti-hemagglutinin antibodies in blood sera at a level of ≥1:40 protects against influenza virus infection. The effectiveness of vaccinations in older people is lower than in young people. After vaccination, most young people achieve this titer, while in the group of older people only ¼ do (Carrat and Valleron, 1994; Glathe and Langer, 1995; Brydak, 2008).

Emerging infectious diseases pose a serious threat to global health security, as exemplified by the recent COVID-19 pandemic. Seasonal influenza virus infections, millions of cases registered every epidemic season and thousands of deaths due to post-influenza complications around the world, as well as the threat of another influenza pandemic and cases of human infection with avian influenza viruses in the past make the fight against influenza one of the public health priorities. Effective control of the threats posed by this disease depends, among others, on effective supervision. A worrying but unfortunately common phenomenon among the society is the disregard of the early symptoms of the disease in particular, when influenza is often confused with the common cold. In many cases, this significantly hinders or completely prevents effective antiviral therapy. Referring to the diagnosis of the influenza virus, the serious consequences resulting from the incorrect diagnosis of symptoms and the implementation of unjustified antibiotic therapy are also worth mentioning. The arising problem is antibiotic resistance, i.e., the lack of sensitivity of bacteria to antibiotics. Due to frequent complications, antibiotic therapy is implemented as part of medical prophylaxis, despite the fact that it is widely known that antibiotics are not used in the prevention of viral infections. The trend of increasing drug resistance of microorganisms, which has been observed in recent years, may lead to a very difficult situation, which is the exhaustion of therapeutic options against infections. The use of antibiotics should therefore be justified by appropriate tests.

Fast and effective diagnosis of patients suspected of having influenza may allow for the earliest possible implementation of treatment and bring about both health-related and economic benefits.

## Conclusion

Based on the assessment of the level of anti-hemagglutinin antibodies in sera collected from patients in seven age groups during the 2021/2022 epidemic season in Poland, the following conclusions can be drawn:

• The study results confirmed the circulation in the population of four antigens of influenza virus strains included in the influenza vaccine for the 2021/2022 epidemic season: A/Victoria/2570/2019 (H1N1) pdm09-like virus, A/Cambodia/e0826360/2020 (H3N2)-like virus, B/Washington/02/2019 (B/Victoria lineage)-like virus and B/Phuket/3073/2013 (B/Yamagata lineage)-like virus.
• Adult patients (of over 14 years of age) had antibodies against hemagglutinin of influenza viruses more often than children (of under 14 years of age) (with the exception of antibodies against A/Victoria/2570/2019 (H1N1) pdm09—here, the level of antibodies in patients from the two above groups was at a similar level).
• Among the tested subjects with antibodies, their titer is on average higher in children under 14 years of age (the exception being antibodies against B/Washington/02/2019 (B/Victoria lineage)—here the level of antibodies in both adult patients over 14 years of age and in children was at a similar level).
• Tested children aged 0–14 years more often achieved a protective level (anti-hemagglutinin antibody titer ≥40) in case of antibodies against A/Victoria/2570/2019 (H1N1) pdm09, A/Cambodia/e0826360/2020 (H3N2) and B/Phuket/3073/2013 (B/Yamagata lineage); the protective level against B/Washington/02/2019 (B/Victoria lineage) was more often achieved by adults over 14 years of age (while as many as 87% of children under 14 years of age did not have anti-hemagglutinin antibodies at all).
• The low percentage of vaccinated persons in particular age groups may indicate that the level of protection obtained may have been the result of a past infection caused by influenza viruses.

## Data Availability

The original contributions presented in the study are included in the article/supplementary material, further inquiries can be directed to the corresponding author.
